# Psychological distress and its association with socio-demographic factors in a rural district in Bangladesh: A cross-sectional study

**DOI:** 10.1371/journal.pone.0212765

**Published:** 2019-03-13

**Authors:** Fakir M. Amirul Islam

**Affiliations:** 1 Department of Statistics, Data Science and Epidemiology; Faculty of Health, Arts and Design; Swinburne University of Technology, Hawthorn VIC, Australia; 2 Organisation for Rural Community Development (ORCD), Dariapur, Narail, Bangladesh; Università degli Studi di Perugia, ITALY

## Abstract

**Background:**

Psychological distress including depression and anxiety are among the most serious causes of morbidity and mortality in Bangladesh. There has been no study in the rural area to report the prevalence of and risk factors for psychological distress. The aim of this study was to estimate the prevalence of and risk factors for psychological distress in a rural district in Bangladesh.

**Methods:**

A total of 2425 adults (1249 women) aged 18–90 years were selected from the Narail upazilla using multi-level cluster random sampling for a cross-sectional study. Psychological distress was assessed using the Kessler 10 items questionnaire. Participants’ socio-demographic status, life style factors and health conditions were also collected. Odds ratios and 95% confidence intervals for binary outcomes and mean changes for continuous outcomes of psychological distress score were computed. Logistic regression and generalized linear model techniques were used for analytical purpose.

**Results:**

The overall prevalence of psychological distress was 52.5%. This proportion included 22.7% people rated as having mild psychological distress, 20.8% moderate and 9.0% severe. The prevalence of moderate (24.7% vs. 17.5%, p<0.001) and severe (16.2% vs. 2.5%, p<0.001) psychological distress was significantly higher in older adults of age 60–90 years than that in younger adults of age 18–59 years. The prevalence of severe psychological distress was higher in females than males and the difference increased with age (vs. (females vs males: 1.9% vs. 1.1% at age of <30 years, 12.2% vs. 10.1% at age between 60–69 years, and 45.5% vs. 25.4% at age of 80 years or older). After multivariate adjustment, compared to degree or equivalent level of education, no education (odds ratio (OR), 1.71, 95% confidence interval (CI), 1.03–2.82) was associated with higher prevalence of any psychological distress in the total sample. Compared to married, psychological distress among widowed older adults was almost five times higher prevalence (OR, 4.89, 95% CI, 2.51–9.55). Socio-economic status showed a U-shaped relationship with the prevalence of psychological distress; being very poor or wealthy was associated with higher prevalence of psychological distress compared to those of moderate socio-economic status. People living in pourashava (semi-urban areas) reported significantly higher prevalence of psychological distress compared to people living in typical rural unions.

**Conclusions:**

In this rural Bangladeshi community, the prevalence of psychological distress was high, especially among older women. Factors including lower level of education, inability to work, and living in semi-urban areas were associated with higher prevalence of psychological distress. Public health programmes should target people in high risk groups to reduce their psychological distress in Bangladesh.

## Introduction

Psychological distress is common across the world [1, 2]. There are currently over 542 million people living with depression or anxiety symptoms, which represents an increase of more than 18% from 2005 to 2015 [3–5]. It is projected that one in four individuals globally will be affected by depressive symptoms at some point in their lifetime [6, 7]. If this depression is present with chronic diseases as co-morbid conditions, it can reduce life expectancy by approximately 20 years [8]. Psychological distress is the presence of a number of depressive symptoms including lack of enthusiasm, feeling hopeless about the future, and anxiety symptoms [9].

The prevalence of psychological distress and its associated factors vary across the globe. A meta-analysis of 174 surveys across 63 countries from 1980 to 2013 comprising 665,433comprised 665433 individuals reported a pooled prevalence of 17.6% (14.7% in men and 19.7% in women) with common mental disorder during 12-months preceding assessments. In addition, 29.2% of respondents had experienced a common mental disorder at some time during their lifetime [10]. A study among South African adults reported 28.4% prevalence of lifetime psychological distress with varying proportions of intensity, such as 10.3% prevalence of moderate levels of distress, 6.4% prevalence of high or very high levels of distress[9]. The lifetime prevalence of psychological distress was 31% among Australian adults with two-thirds reporting moderate and one-third reporting high level of psychological distress [11]. Bangladesh is one of the most densely populated countries in the world, with a population of approximately 163 million people [12]. Depression and anxiety are among the most serious causes of morbidity and mortality in Bangladesh [13–16]. An urban tertiary hospital based study reported 47% of patients with stroke and 54% of patients with cancer had severe depression in Bangladesh [17]. Despite the adverse impacts of such mental health conditions, they receive little attention in most of the low and middle-income countries, including Bangladesh, and hence, treatment of such conditions is not considered a national health priority [18, 19]. In terms of service delivery, mental health services are almost non-existent at primary care level throughout the country and the referral to hospital is mostly a long delay. A study conducted in a rural area reported that 65% of patients with the mental health condition were referred to the hospital 3 months to several years after onset of the disorder [20, 21]. In several studies, the prevalence of and risk factors for psychological distress both in urban and rural areas, both at community and facility based settings in Bangladesh, was found to vary between 6.5 and 31.0% [20].

All studies of psychological distress or depression and anxiety and their associated risk factors were conducted over a decade ago, despite significant changes in socio-demographic factors and life expectancy. For example, life expectancy in Bangladesh was 44.9 years in 1975, 58.1 years in 2000 [22] and currently 72 years in 2018 [23]. Therefore, there is a need to accurately investigate the current prevalence of and risk factors for psychological distress. It is also important to identify the groups at most risk of psychological distress at a population level in order to develop appropriate intervention and prevention strategies. The need was also highlighted by Hossain et al. in their systematic review [20]. The current study aims to estimate the prevalence of psychological distress, its severity and the associated factors, by conducting across-sectional study in people with a wide age distribution in a rural district in Bangladesh.

## Materials and methods

### Study location

Data were collected from the Narail upazila, which is considered to be reflective of typical rural demography in Bangladesh. Bangladesh **i**s divided into 8 administrative divisions, each of which is divided into a number of districts and thus there are 64 districts, or zila, in total. For the purposes of local government, each district/zila is divided into a number of upazila. There are 493 upazila in total in Bangladesh with 163 million people [24]. Narail district is located approximately 150 km south-west of Dhaka, the capital city of Bangladesh, with a population of 272,872. Narail has an estimated population density of 722 people per km^2^, which is comparable to the national rural population density of 873 people per km^2^[24].

### Sample size and statistical power

The sample size was calculated based on severe depression in adults of age 18 to 59 years and older adults of age 60 to 90 years. Prior prevalence of severe depression was 6.5% in adults and 21% in older adults [20]. With the assumption of margin of error of 3% in prevalence for adults, and of 5% in older adults, with a significance level of 0.05 and statistical power above 80% a required sample size of 1283 was needed for adults and 1128 for the older adults. In the current study, we ended up recruiting 1278 adults and 1147 older adults, i.e. 2425 total participants. Since the sample size is large enough to detect a minimum prevalence of 6.5%, the estimated sample size is large enough for any prevalence larger than 6.5%, as well as both in adults and older adults group, and by gender. Exclusion criteria were age <18 years or >90 years, and any severe illness preventing participants from participating in the study.

### Sampling frame and recruitment

Narail upazilla consists of 13 rural unions, and an urban city known as Narail paurashova, which consists of 9 wards. Each union consists of 15–20 villages and a ward consists of 8–12 mahallas or para and each village or mahalla consists of 200 to 500 households depending on the size of the villages/mahallas. Three from 13 rural unions and one from 9 urban wards were randomly selected at cluster level 1. Two to three villages or mahallas from each selected union or ward were randomly selected at level 2. The participants from the village or mahalla were not selected at random but prioritisation was given in recruiting older adults first. The recruitment started from a corner of a village and continued until the recruitment of a maximum of 250 participants was reached for a large village where the number of eligible participants were greater than 250. In case of fewer than 250 households in a village, the recruitment continued to the adjacent village to reach the number to 250. Data were collected by three teams, each of which had 3 members. The team members participated in an intensive 2-day training programme in Narail before the commencement of data collection. The interviewers were instructed to interview an older adult first. If none were available in this subgroup, the interviewers approached an adult person of that household. Again, if there was more than one male or female adult in the same household, one individual was selected, based on who was born closer to January. However, to maintain an approximately equal number of males and female participants, one female was interview immediately after a male participant. The K10 was translated into Bengali independently by two bilingual translators including a medical practitioner with experience in public health. The K10 questionnaire was translated using back-translation techniques [25]. We have reported the study sample, recruitment strategy, and data collection previously [26].

### Outcome variables

The Kessler 10 items questionnaire [27] was used for measuring psychological distress, which is the main outcome variable for this study. The Kessler 10 (K10) items is based on 10 items that measure the frequency of non-specific psychological distress symptoms during the past four weeks. Respondents were asked, “During the past four weeks, how often did you feel: 1) tired out for no good reason; 2) nervous; 3) so nervous that nothing could calm you down; 4) hopeless; 5) restless or fidgety; 6) so restless you could not sit still; 7) sad or depressed; 8) so depressed that nothing could cheer you up; 9) everything was an effort; 10) worthless.” Items were rated on a five-point ordinal scale― none of the time (score 1), a little of the time (score 2), some of the time (score 3), most of the time (score 4), and all of the time (score 5). The total K10 score for each respondent was calculated by summing all 10 items, which then ranged from a minimum of 10 to a maximum of 50. The total scores were then categorised into: no psychological distress (10 to 19), mild (20 to 24), moderate (25 to 29) and severe psychological distress (30 to 50) as per Andrews[28] and Kessler [27]. The psychometric properties of the K10 questionnaire were investigated using a Rasch analysis and proposed to be a modified version of seven items with 1 to 4 categories [29]. The total K7 score for each respondent was calculated by summing all 7 items, which then ranged from a minimum of 7 and a maximum of 28 to investigate the robustness of the findings from the K10.

### Independent variables

Socio-demographic factors including age, gender and level of education were collected. Level of education was categorised into: no schooling, primary school level of education (grade 1 to 5), secondary school level of education (grade 6 to 10) and school secondary certificate (SSC) or above. Socio-economic status (SES) was assessed according to Cheng et al.[30] asking whether "over the last twelve months, in terms of household food consumption, how would you classify your socio-economic status?" with the possible answers: (i) insufficient funds for the whole year, (ii) insufficient funds some of the time, (iii) neither deficit nor surplus (balance), and (iv) sufficient funds most of the time. Data on current occupation (e.g., student, housework, daily labour, government or non-government job, business, and unable to work or retired), marital status, current health problems (yes or no), number of health problems, and medication use were also collected during the interview.

### Statistical analysis

Participant characteristics including age, gender and level of education are presented for adults and older adults using descriptive statistics. We used binary logistic regression models to estimate the odds ratio (OR) and 95% confidence intervals (CI) for any psychological distress. In this case, participants were categorised into (1) no psychological distress (scores of 19 and lower) and (2) any level of psychological distress (scores of 20 and above) from the total score of 50 in association with categorical responses of the independent variables (e.g., level of education). We used multinomial logistic regression techniques to determine the odds of increasing the severity of psychological distress (normal, mild, moderate and severe) associated with different binary, multi-category or incremental categories for ordinal cut-off of quantitative variables. We also used a generalised linear model in presenting mean difference and 95% confidence interval for psychological distress score on a continuous scale ranging from 10 to 50 units for K10 items and 7 to 28 units for modified K7 items in association with categorical responses of the independent variables. All models were adjusted for age, gender, level of education, marital status, socio-economic status, occupation, smoking status, medical condition and geographic location. Statistical software SPSS (SPSS Inc, version 24) was used for the analysis.

### Ethical approval

We conducted the research following the tenets of the Declaration of Helsinki. Human Ethics Approval was received from the Swinburne University of Technology Human Ethics Committee (SHR Project 2015/065). We obtained written consent from the participants who were able to sign and a finger print was obtained from those who were unable to sign. In the case of finger print consent, the data collector provided a counter signature for the participants. Participants were informed of their right to withdraw from the study at any stage if they wished.

## Results

Of 2425 participants, 51.5% were female (52.8% in adults and 50% in older adults). In comparison of socio-demographic factors between adults and older adults, 13.4% vs. 43.6% had no schooling, 9% vs. 30% had house duties, 16% vs. 7.9% were labourers, 91% vs. 68% were married, 3% vs. 32% were widowed, 26% vs. 28.6% were tobacco smokers and 11.0% vs. 37.9% consumed smokeless tobacco (SLT), respectively (Table 1).

**Table 1:** Sociodemographic characteristic of adults and older adults in a rural district in Bangladesh.

Characteristic	Total, N = 2425	Adults (18–59 years), N = 1278 (52.7%)	Older adults (60–90 years), N = 1147 (47.3%)
Gender	Number (%)	Number (%)	Number (%)
Female	1249 (51.5)	675 (52.8)	574 (50)
Male	1176 (48.5)	603 (47.2)	573 (50)
Level of education (in years)			
No education	671 (27.7)	171 (13.4)	500 (43.6)
Primary (1–5)	946 (39.0)	510 (39.9)	436 (38.0)
Secondary (6–9)	327 (13.5)	238 (18.6)	89 (7.8)
SSC or HSC Pass (10–12)	385 (15.9)	295 (23.1)	90 (7.8)
Degree or equivalent (13–16)	96 (4.0)	64 (5.0)	32 (2.8)
Socio-economic Condition:			
Insufficient funds most of the time	367 (15.1)	172 (13.5)	195 (17.0)
Insufficient funds some of the time	781 (32.2)	400 (31.3)	381 (33.2)
Balance	1037 (43.0)	582 (45.5)	455 (39.7)
Sufficient funds most of the time	240 (9.9)	124 (9.7)	116 (10.1)
Occupation			
Housework	970 (40)	629 (49)	341 (30)
Student	45 (1.9)	40 (3.1)	5 (0.4)
Farming	217 (8.9)	130 (10.2)	87 (7.6)
Labourers	297 (12)	206 (16.1)	91 (7.9)
Business, government or non-government job	359 (14.9)	262 (20.5)	97 (88.5)
Retired or unable to work	537 (22.1)	11 (0.9)	526 (46)
Marital status			
Married	1937 (80.0)	1163 (91.0)	774 (68)
Widow	405 (17.0)	38 (3.0)	367 (32)
Unmarried/never married	78 (3.2)	73 (5.7)	5 (0.4)
Divorce/separation	5 (0.2)	4 (0.3)	1 (0.1)
Smoking status			
Never smoker	1180 (49.0)	800 (62.9)	380 (33.5)
Ever of current tobacco smoker	656 (27.3)	331 (26.0)	325 (28.6)
Smokeless tobacco consumption	570 (23.7)	140 (11.0)	430 (37.9)
Any medical condition			
No	1063 (43.9)	803 (62.8)	260 (22.7)
Yes	1361(56.1)	476 (37.2)	885 (77.3)
Geographic location			
Urban pouroshova	685 (28.2)	388 (56.6)	297 (43.4)
Rural Banshgram	656 (27.1)	381 (58.1)	275 (41.9)
Rural Tularampur	501 (20.7)	209 (41.7)	292 (58.3)
Rural Vodrobilla	583 (24.0)	301 (51.6)	282 (48.4)

**Fig 1:**
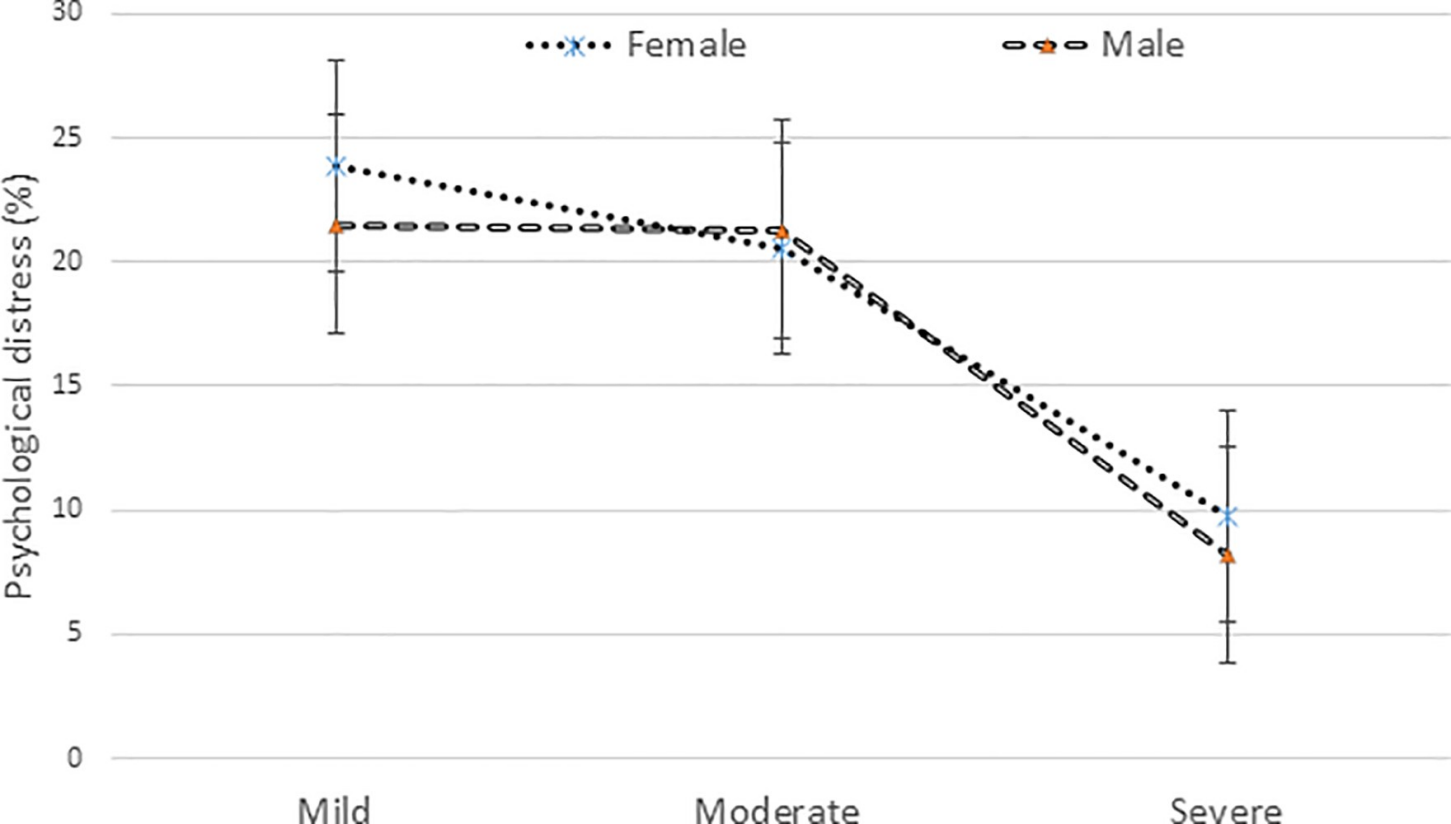
Different levels of psychological distress in males and females in total sample.

**Fig 2:**
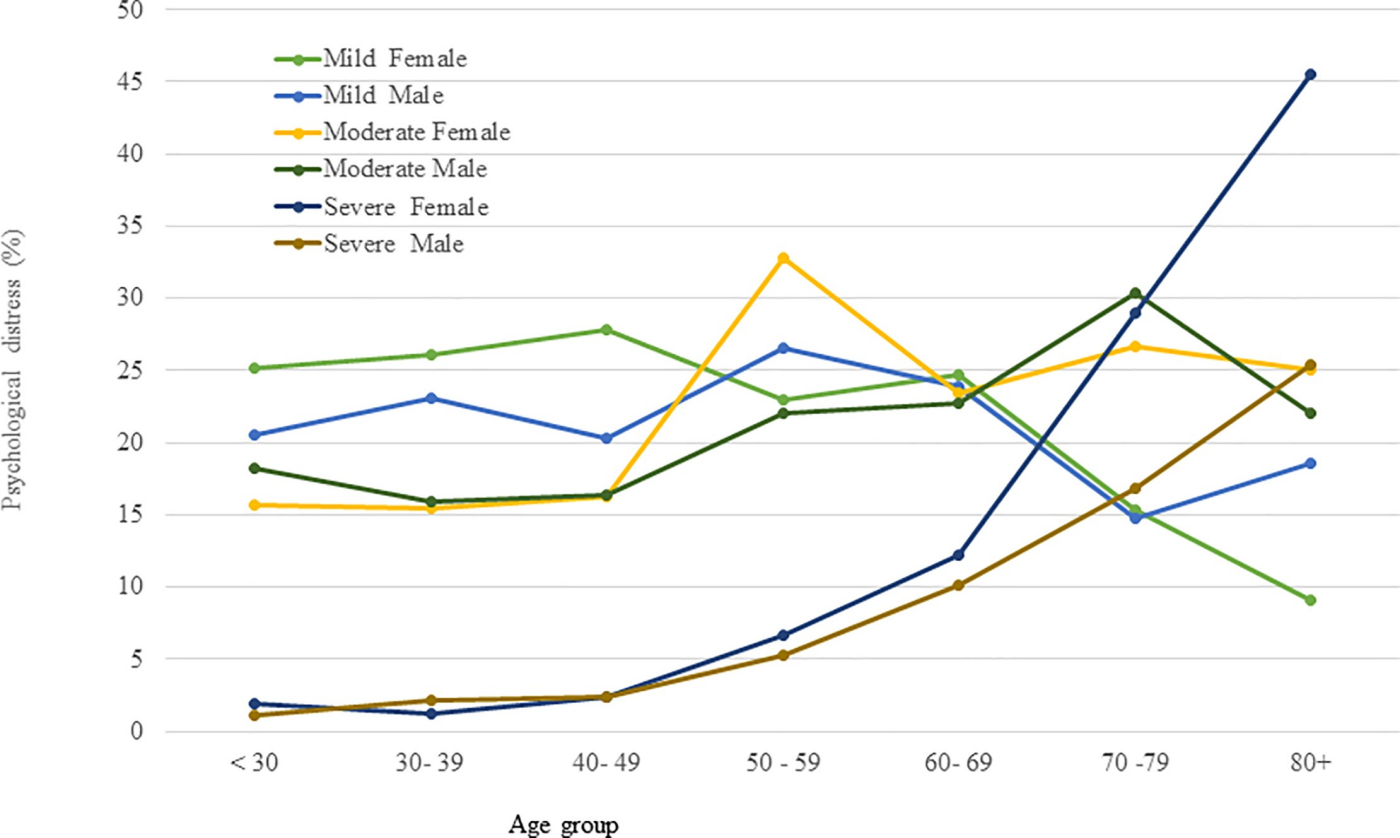
Different levels of psychological distress in males and females by age groups.

The prevalence of any level of psychological distress was 52.5% in the total sample, with a significant difference between adults and older adults (44.5% in adults vs. 61.8% in older adults, p<0.001). There was no significant difference in any psychological distress or severity of psychological distress between males and females (Fig 1). However, after the age of 60 years, the prevalence of severe psychological distress was significantly higher in females than in males; in contrast, mild psychological distress was higher in males (Fig 2). The prevalence of any psychological distress was higher in people with no education (60.1%), being widowed (70.1%), unable to work or retired (62.0%), insufficient funds most of the time (59.9%) or suffering from any health problem (58.3%) than their counterparts.

**Table 2** shows individual risk factor associations for psychological distress after adjustment for age, gender, level of education, marital status, socio-economic status, occupation, smoking status, medical condition and geographic location. Compared to adults of age 18–59 years, older adults of age 60–90 years reported 50% higher prevalence of any psychological distress (odds ratio (OR) 1.50, 95% confidence interval (CI) 1.19–1.88). There was a U-shape relationship observed between the socio-economic status and any psychological distress. For example, having insufficient funds most of the time (very poor) (OR 1.65, 95% CI 1.25–2.17) or sufficient funds most of the time (wealthy) (OR 1.89, 95% CI 1.34–2.64) were associated with higher prevalence of any psychological distress compared to those with insufficient funds some of the time (poor or middle class)

**Table 2:** Association of socio-demographic factors with any psychological distress in the total participants using binary regression model.

	No at risk	Normal, n (%)	Any PD, n (%)	OR (95% CI)*
Total	2425	1149 (47.4)	1276 (52.5)	
Age : Adult	1279	711 (55.6)	568 (44.4)	1.0
Elderly	1146	438 (38.2)	708 (61.8)	1.50 (1.19, 1.88)
Age group: 18–29	247	144 (58.3)	103 (41.7)	1.0
30–39	423	245 (57.9)	178 (42.1)	1.05 (0.73, 1.51)
40–49	416	238 (57.2)	178 (42.8)	1.04 (0.72, 1.52)
50–59	193	84 (43.5)	109 (56.5)	1.58 (1.01, 2.47)
60–69	728	301 (41.3)	427 (58.7)	1.57 (1.07, 2.31)
70–79	315	108 (34.3)	207 (65.7)	2.88 (1.78, 4.68)
80 and above	103	29 (28.2)	74 (71.8)	3.58 (1.89, 6.78)
Sex: Female	1238	567 (45.8)	671 (54.2)	1.0
Male	1187	582 (49.0)	605 (51.0)	0.94 (0.67, 1.33)
Education: Degree or equivalent	96	48 (50.0)	48 (50.0)	1.0
SSC or HSC pass	385	212 (55.1)	173 (44.9)	1.09 (0.67, 1.76)
Secondary (6–10)	327	180 (55.0)	147 (45.0)	1.27 (0.76, 2.12)
Primary (1–5)	946	441 (46.6)	505 (53.4)	1.53 (0.95, 2.47)
No Education	671	268 (39.9)	403 (60.1)	1.71 (1.03, 2.83)
Marital status: Married	1937	975 (50.3)	962 (49.7)	1.0
Widow	405	121 (29.9)	284 (70.1)	1.76 (1.31, 2.36)
unmarired/never married	78	49 (62.8)	29 (37.2)	0.58 (0.3, 1.11)
Housework	976	474 (48.6)	502 (51.4)	1.0
Occupation: Student	45	20 (44.4)	25 (55.6)	2.69 (1.17, 6.16)
Farming	217	105 (48.4)	112 (51.6)	1.22 (0.77, 1.92)
Labour	220	115 (52.3)	105 (47.7)	0.94 (0.59, 1.49)
Business, govt. or non-govt job	419	223 (53.2)	196 (46.8)	0.86 (0.58, 1.29)
unable to work or retired	534	203 (38.0)	331 (62.0)	1.09 (0.76, 1.56)
SES: insufficient funds most of the time	367	147 (40.1)	220 (59.9)	1.65 (1.25, 2.17)
Insufficient funds some of the time	781	448 (57.4)	333 (42.6)	1.0 (ref)
Balance	1037	456 (44.0)	581 (56.0)	1.74 (1.40, 2.15)
Sufficient funds most of the time	240	98 (40.8)	142 (59.2)	1.89 (1.34, 2.64)
Health Problems: No	1063	581 (54.7)	482 (45.3)	1.0 (ref)
Yes	1361	568 (41.7)	793 (58.3)	1.35 (1.04, 1.74)
Medication use: No	1527	787 (51.5)	740 (48.5)	1.0
Yes	898	362 (40.3)	536 (59.7)	1.14 (0.89, 1.47)
Smoking: Never smoker	1180	617 (52.3)	563 (47.7)	1.0
Current or past smoker	656	281 (42.8)	375 (57.2)	1.42 (1.12, 1.81)
Smokeless tobacco	570	236 (41.4)	334 (58.6)	1.11 (0.88, 1.41)
Geographic location: Urban pouroshova	685	261 (38.1)	424 (61.9)	1.0
Rural Banshgram	656	273 (41.6)	383 (58.4)	0.76 (0.59, 0.97)
Rural Tularampur	501	325 (64.9)	176 (35.1)	0.25 (0.19, 0.33)
Rural Vodrobilla	583	290 (49.7)	293 (50.3)	0.47 (0.36, 0.6)

OR (95% CI)* adjusted for age, gender, level of education, marital status, socio-economic condition, occupation, smoking status, medical condition and geographic location.

The association of any psychological distress in association with risk factors for adults, older adults and for males and females are shown in Table 3. Having insufficient funds most of the time was associated with higher prevalence of psychological distress both in adults and older adults. However, having sufficient funds most of the time was only associated with higher prevalence of psychological distress in older adults (OR 4.06, 95% CI 2.37–6.95). Compared to married, being widowed was significantly associated with higher prevalence of any psychological distress in older adults, especially among older males (OR 4.89, 95% CI 2.51–9.55). Compared to the urban pourashava, people living in rural unions reported lower prevalence of psychological distress. Although no education was associated with higher prevalence of any psychological distress in the total sample, it was no longer significant after stratification for adults and older adults, or for gender.

**Table 3:** Association of socio-demographic factors with any psychological distress for adults and older adults, and for males and females.

	Adults, N = 1278		Older adults, N = 1147		Females, N = 1238		Males, N = 1187	
Any psychological distress, N (%)	568 (44.4)		708 (61.8)		671 (54.4)		605 (51.0)	
	n (%)	OR (95% CI)*	n (%)	OR (95% CI)*	n (%)	OR (95% CI)*	n (%)	OR (95% CI)*
Adults					306 (45.7)	1.0	262 (43.0)	1.0
Older adults					365 (64.3)	1.46 (1.04,2.05)	343 (59.3)	1.56 (1.13, 2.16)
Sex: Female	306 (45.7)	1.0	365 (64.3)	1.0				
Male	262 (43.0)	0.72 (0.37, 1.44)	343 (59.3)	1.12 (0.74, 1.69)				
Education: Degree or equivalent	33 (51.6)	1.0	15 (46.9)	1.0	15 (57.7)	1.0	33 (47.1)	1.0
SSC or HSC pass	131 (44.4)	0.94 (0.51, 1.71)	42 (46.7)	1.10 (0.47, 2.58)	68 (42.8)	0.68 (0.26, 1.79)	105 (46.5)	1.38 (0.76, 2.48)
Secondary (6–10)	97 (40.8)	1.19 (0.62, 2.3)	50 (56.2)	1.64 (0.69, 3.91)	79 (44.1)	0.82 (0.31, 2.21)	68 (45.9)	1.58 (0.82, 3.03)
Primary (1–5)	222 (43.4)	1.22 (0.66, 2.29)	283 (65.1)	2.19 (1.0, 4.81)	265 (53.9)	0.96 (0.37, 2.53)	240 (52.9)	1.92 (1.07, 3.45)
No Education	85 (49.7)	1.67 (0.83, 3.36)	318 (63.6)	2.16 (0.97, 4.81)	244 (63.9)	1.29 (0.47, 3.5)	159 (55)	1.76 (0.95, 3.26)
Marital status: Married	520 (44.7)	1.0	442 (57.2)	1.0	437 (49.8)	1.0	525 (49.6)	1.0
Widow	20 (52.6)	1.29 (0.61, 2.73)	264 (71.9)	2.02 (1.44, 2.82)	225 (67.6)	1.32 (0.92, 1.88)	59 (81.9)	4.89 (2.51, 9.55)
unmarired/never married	27 (37)	0.6 (0.29, 1.23)	2 (40)	0.65 (0.1, 4.34)	8 (34.8)	0.16 (0.03, 0.86)	21 (38.2)	0.8 (0.39, 1.63)
Occupation: Homemaker	287 (45.4)	1.0	215 (62.5)	1.0	496 (51.7)	1.0	6 (35.3)	1.0
Student	20 (50)	2.16 (0.81, 5.75)	5 (100)	—	14 (56)	7.56 (1.42, 40.28)	11 (55)	7.31 (1.48, 36.14)
Farmer	54 (41.5)	1.46 (0.66, 3.26)	58 (66.7)	1.47 (0.76, 2.83)	0	0	112 (52.3)	5.12 (1.52, 17.32)
Labour	80 (48.8)	1.57 (0.71, 3.46)	25 (44.6)	0.56 (0.27, 1.18)	0	0	105 (48.2)	3.31 (0.98, 11.21)
Business or any govt/non-govt job	120 (41.1)	0.99 (0.48, 2.04)	76 (59.8)	0.93 (0.53, 1.62)	13 (56.5)	1.05 (0.39, 2.8)	183 (46.2)	3.25 (0.99, 10.75)
unable to work or retired	4 (36.4)	0.86 (0.18, 4.21)	327 (62.5)	0.9 (0.6, 1.34)	147 (65.9)	1.08 (0.71, 1.66)	184 (59.2)	3.92 (1.17, 13.19)
SES: insufficient funds most of the time	85 (49.4)	1.51 (1.0, 2.28)	135 (69.2)	1.77 (1.19, 2.63)	127 (64.8)	1.69 (1.14, 2.50)	93 (54.4)	1.66 (1.10, 2.49)
Insufficient funds some of the time	132 (32.9)	1.0 (ref)	201 (52.9)	1.0 (ref)	175 (43.3)	1.0 (ref)	158 (41.9)	1.0 (ref)
Balance	298 (51.2)	1.58 (1.15, 2.17)	283 (62.2)	1.78 (1.30, 2.45)	299 (57.9)	1.91 (1.42, 2.58)	282 (54.1)	1.59 (1.17, 2.18)
Sufficient funds most of the time	53 (42.7)	0.92 (0.56, 1.51)	89 (76.7)	4.06 (2.37, 6.95)	70 (57.4)	1.63 (1.00, 2.63)	72 (61)	2.20 (1.35, 3.60)
Health Problems: No	342 (42.6)	1.0	140 (53.8)	1.0	233 (45.2)	1.0	249 (45.5)	1.0
Yes	226 (47.5)	0.98 (0.65, 1.47)	567 (64.1)	1.75 (1.2, 2.55)	438 (60.7)	1.52 (1.05, 2.18)	355 (55.6)	1.22 (0.84, 1.77)
Medication use: No	403 (41.8)	1.0	337 (59.9)	1.0	383 (49.7)	1.0	357 (47.2)	1.0
Yes	165 (52.4)	1.8 (1.15, 2.8)	371 (63.6)	0.86 (0.63, 1.17)	288 (61.7)	1.07 (0.76, 1.51)	248 (57.5)	1.2 (0.83, 1.73)
Smoking: Never smoker	358 (44.8)	1.0	205 (53.9)	1.0	361 (48.1)	1.0	202 (47)	1.0
Current or past smoker	149 (45)	1.28 (0.89, 1.82)	226 (69.5)	1.76 (1.23, 2.5)	87 (77)	1.96 (1.15, 3.35)	288 (53)	1.39 (1.03, 1.86)
Smokeless tobacco	60 (42.9)	0.84 (0.55, 1.29)	274 (63.7)	1.28 (0.94, 1.75)	223 (59.5)	1.06 (0.77, 1.44)	111 (56.9)	1.28 (0.87, 1.9)
Geographic Location: Urban Pourashava	239 (61.6)	1.0	185 (62.3)	1.0	213 (61.6)	1.0	211 (62.2)	1.0
Banshgram	201 (52.8)	0.62 (0.44, 0.86)	182 (66.2)	0.92 (0.62, 1.36)	212 (62.9)	0.96 (0.67, 1.36)	171 (53.6)	0.57 (0.4, 0.82)
Tularampur	9 (4.3)	0.02 (0.01, 0.05)	167 (57.2)	0.75 (0.52, 1.09)	87 (34.5)	0.28 (0.19, 0.42)	89 (35.7)	0.21 (0.14, 0.31)
Vodrobilla	119 (39.5)	0.34 (0.24, 0.48)	174 (61.7)	0.69 (0.47, 1.02)	159 (52.5)	0.59 (0.41, 0.84)	134 (47.9)	0.33 (0.23, 0.49)

* OR (95% CI) adjusted for age, gender, level of education, marital status, socio-economic condition, occupation, smoking status and medical condition and geographic location.

The prevalence of mild psychological distress (adults vs. older adults: 24.4% vs. 20.9%) was higher in adults, but the prevalence of moderate (17.5% vs. 24.7%) and severe psychological distress (2.5% vs. 16.2%) was significantly higher in older adults. The prevalence of severe psychological distress was higher in older adults, especially in people with no education (21.4%), widowed (24.8%) and unable to work or retired (20.3%) compared to the overall 16.2% in the older adults (Table 4).

**Table 4:** Any psychological distress and it’s severity by socio-demographic factors of the total participants and by adults and older adults.

	Total				Adults				Older adults			
	No at risk	Mild, n(%)	Moderate, n(%)	Severe, n(%)	No at risk	Mild, n(%)	Moderate, n(%)	Severe, n(%)	No at risk	Mild, n(%)	Moderate, n(%)	Severe, n(%)
Total	2425	551 (22.7)	507 (20.9)	218 (9.0)	1279	312(24.4)	224(17.5)	32(2.5)	1146	239(20.9)	283(24.7)	186(16.2)
Sex: Female	1238	296 (23.9)	254 (20.5)	121 (9.8)	670	175 (26.1)	116 (17.3)	15 (2.2)	568	121 (21.3)	138 (24.3)	106 (18.7)
Male	1187	255 (21.5)	253 (21.3)	97 (8.2)	609	137 (22.5)	108 (17.7)	17 (2.8)	578	118 (20.4)	145 (25.1)	80 (13.8)
Education: No Education	671	116 (17.3)	175 (26.1)	112 (16.7)	171	35 (20.5)	45 (26.3)	5 (2.9)	500	81 (16.2)	130 (26)	107 (21.4)
Primary (1–5)	946	255 (27)	176 (18.6)	74 (7.8)	511	145 (28.4)	65 (12.7)	12 (2.3)	435	110 (25.3)	111 (25.5)	62 (14.3)
Secondary (6–10)	327	76 (23.2)	58 (17.7)	13 (4)	238	53 (22.3)	40 (16.8)	4 (1.7)	89	23 (25.8)	18 (20.2)	9 (10.1)
SSC or HSC pass	385	83 (21.6)	76 (19.7)	14 (3.6)	295	64 (21.7)	58 (19.7)	9 (3.1)	90	19 (21.1)	18 (20)	5 (5.6)
Degree or equivalent	96	21 (21.9)	22 (22.9)	5 (5.2)	64	15 (23.4)	16 (25)	2 (3.1)	32	6 (18.8)	6 (18.8)	3 (9.4)
Marital status: Married	1937	451 (23.3)	387 (20)	124 (6.4)	1164	284 (24.4)	207 (17.8)	29 (2.5)	773	167 (21.6)	180 (23.3)	95 (12.3)
Widow	405	83 (20.5)	109 (26.9)	92 (22.7)	38	12 (31.6)	7 (18.4)	1 (2.6)	367	71 (19.3)	102 (27.8)	91 (24.8)
unmarired/never married	78	17 (21.8)	10 (12.8)	2 (2.6)	73	16 (21.9)	9 (12.3)	2 (2.7)	5	1 (20)	1 (20)	0 (0)
Occupation: Student	45	14 (31.1)	10 (22.2)	1 (2.2)	40	12 (30)	7 (17.5)	1 (2.5)	5	2 (40)	3 (60)	0 (0)
Housewives	976	249 (25.5)	191 (19.6)	62 (6.4)	632	165 (26.1)	108 (17.1)	14 (2.2)	344	84 (24.4)	83 (24.1)	48 (14)
land owner	217	49 (22.6)	51 (23.5)	12 (5.5)	130	23 (17.7)	28 (21.5)	3 (2.3)	87	26 (29.9)	23 (26.4)	9 (10.3)
Labour	220	47 (21.4)	49 (22.3)	9 (4.1)	164	39 (23.8)	35 (21.3)	6 (3.7)	56	8 (14.3)	14 (25)	3 (5.4)
Business	282	74 (26.2)	40 (14.2)	19 (6.7)	195	52 (26.7)	25 (12.8)	1 (0.5)	87	22 (25.3)	15 (17.2)	18 (20.7)
Govt. or non-govt job	137	29 (21.2)	28 (20.4)	6 (4.4)	97	20 (20.6)	18 (18.6)	4 (4.1)	40	9 (22.5)	10 (25)	2 (5)
unable to work or retired	534	88 (16.5)	135 (25.3)	108 (20.2)	11	1 (9.1)	1 (9.1)	2 (18.2)	523	87 (16.6)	134 (25.6)	106 (20.3)
SES: Very poor	367	111 (30.2)	75 (20.4)	34 (9.3)	172	61 (35.5)	20 (11.6)	4 (2.3)	195	50 (25.6)	55 (28.2)	30 (15.4)
Poor	781	139 (17.8)	121 (15.5)	73 (9.3)	401	77 (19.2)	48 (12)	7 (1.7)	380	62 (16.3)	73 (19.2)	66 (17.4)
Middle class	1037	237 (22.9)	254 (24.5)	90 (8.7)	582	149 (25.6)	134 (23)	15 (2.6)	455	88 (19.3)	120 (26.4)	75 (16.5)
Rich	240	64 (26.7)	57 (23.8)	21 (8.8)	124	25 (20.2)	22 (17.7)	6 (4.8)	116	39 (33.6)	35 (30.2)	15 (12.9)
Health Problems: No	1063	208 (19.6)	232 (21.8)	42 (4)	803	165 (20.5)	162 (20.2)	15 (1.9)	260	43 (16.5)	70 (26.9)	27 (10.4)
Yes	1361	343 (25.2)	274 (20.1)	176 (12.9)	476	147 (30.9)	62 (13)	17 (3.6)	885	196 (22.1)	212 (24)	159 (18.0)
Medication use: No	1527	312 (20.4)	314 (20.6)	114 (7.5)	964	204 (21.2)	179 (18.6)	20 (2.1)	563	108 (19.2)	135 (24)	94 (16.7)
Yes	898	239 (26.6)	193 (21.5)	104 (11.6)	315	108 (34.3)	45 (14.3)	12 (3.8)	583	131 (22.5)	148 (25.4)	92 (15.8)
Smoking: Never smoker	1180	277 (23.5)	218 (18.5)	68 (5.8)	800	204 (25.5)	139 (17.4)	15 (1.9)	380	73 (19.2)	79 (20.8)	53 (13.9)
current or past smoker	656	138 (21)	165 (25.2)	72 (11)	331	70 (21.1)	64 (19.3)	15 (4.5)	325	68 (20.9)	101 (31.1)	57 (17.5)
Smokeless tobacco	570	133 (23.3)	123 (21.6)	78 (13.7)	140	37 (26.4)	21 (15)	2 (1.4)	430	96 (22.3)	102 (23.7)	76 (17.7)
Geographic Location: Urban Pourashava	685	144(21)	219(32)	61(8.9)	388	100(25.8)	118(30.4)	21(5.4)	297	44(14.8)	101(34)	40(13.5)
Banshgram	656	192(29.3)	131(20)	60(9.1)	381	122(32)	70(18.4)	9(2.4)	275	70(25.5)	61(22.2)	51(18.5)
Tularampur	501	58(11.6)	43(8.6)	75(15)	209	7(3.3)	0()	2(1)	292	51(17.5)	43(14.7)	73(25)
Vodrobilla	583	157(26.9)	114(19.6)	22(3.8)	301	83(27.6)	36(12)	0()	282	74(26.2)	78(27.7)	22(7.8)

Following the multivariate adjustment and using the multinomial logistic regression techniques, older age, having insufficient funds most of the time, suffering from any health problem and ever smoking were associated with the severity of psychological distress in total sample as well as in both adults and older adults. For example, among males, having insufficient funds most of the time was associated with a 153% higher prevalence of moderate psychological distress (OR 2.53, 95% CI 1.79, 3.57) and 174% higher prevalence of severe psychological distress (OR 2.74, 95% CI 1.22, 6.15) compared to people who had insufficient funds some of the time. The associations of SES with severity of psychological distress in older adults were similar to those observed in adults. Being widowed was associated with higher prevalence of moderate (OR 1.92, 95% CI 1.31, 2.80) and severe (OR 2.73, 95% CI 1.79, 4.16) psychological distress in older adults but not in adults. For older adults, smoking was associated with higher prevalence of mild (OR 1.56, 95% CI 1.11, 2.19), moderate (OR 1.71, 95% CI 1.22, 2.36) and severe (OR 1.57, 95% CI 1.07, 2.30) psychological distress but not for adults (Table 5).

**Table 5:** Association of socio-demographic and other characteristics with the severity of psychological distress in a multi-nominal logistic regression model by adults and older adults.

	Total				Adult			Older adult		
	Normal	Mild	Moderate	Severe	Mild	Moderate	Severe	Mild	Moderate	Severe
		OR (95% CI) †	OR (95% CI) †	OR (95% CI) †	OR (95% CI) †	OR(95% CI) †	OR (95% CI) †	OR(95% CI) †	OR (95% CI) †	OR (95% CI) †
Older adults vs adults	1.0	1.24 (1.01, 1.53)	2.05(1.66, 2.54)	9.44 (6.37, 14.0)**						
Male vs. Female	1.0	0.84 (0.69, 1.03)	0.97 (0.79, 1.2)	0.78 (0.58, 1.05)	0.82(0.63, 1.07)	0.98(0.72, 1.32)	1.19(0.59, 2.42)	0.84(0.61, 1.16)	0.91(0.67, 1.22)	0.65(0.46, 0.92)
‡Education level	1.0	1.29 (0.98, 1.69)	0.65 (0.5, 0.84)	0.57 (0.4, 0.8)**	1.07(0.69, 1.64)	0.43(0.28, 0.66)	0.74(0.27, 2.05)**	1.46(1.02, 2.09)	0.83(0.6, 1.16)	0.62(0.42, 0.91)**
⁋SES	1.0	1.38 (1.11, 1.72)	2.11(1.68, 2.67)	1.82 (1.31, 2.52)**	1.30(0.97, 1.73)	2.53(1.79, 3.57)	2.74(1.22, 6.15)**	1.41(1.0, 1.97)	1.73(1.25, 2.39)	1.62(1.11, 2.35)*
Widowed vs. married	1.0	1.33 (0.94, 1.88)	1.74 (1.25, 2.44)	2.57 (1.73, 3.81)**	1.34(0.63, 2.86)	1.37(0.54, 3.42)	1.11(0.14, 9.07)	1.39(0.93, 2.07)	1.92(1.31, 2.8)	2.73(1.79, 4.16)**
Health problem vs. no problem	1.0	1.74 (1.38, 2.19)	0.9 (0.71, 1.15)	1.91 (1.29, 2.83)**	1.71(1.29, 2.27)	0.73(0.51, 1.03)	1.99(0.94, 4.24)**	1.81(1.21, 2.71)	1.12(0.79, 1.6)	2.02(1.26, 3.24)*
Ever smoker vs. never smoker	1.0	1.17 (0.93, 1.47)	1.32 (1.04, 1.67)	1.5 (1.07, 2.11)*	0.90(0.65, 1.25)	1.04(0.72, 1.52)	2.45(1.05, 5.72)	1.56(1.11, 2.19)	1.71(1.22, 2.36)	1.57(1.07, 2.3)*

†Odds Ratio (95% Confidence Interval (CI)) adjusted for variables included in the model

‡Education level was not adjusted for SES and vice versa.

⁋Insufficient funds some of the time is the reference group

*indicates a significant trend with p≤0.05 and

** indicates p≤0.001.

In a generalised linear model adjusting for similar risk factors, older adults, no education, widowed, any health problem, current or past smoker and the people living in the urban pourashava were associated with higher psychological distress scores, and having insufficient funds some of the time was associated with lower psychological distress scores. The results were consistent for scores computed from the K10 items and the validated K7 items, in the total sample, and in adults and older adults as well (Table 6).

**Table 6:** Effect size (change in psychological distress score) in total sample, by adults and older adults for K10 and validated K7 scale.

		K10: Total	K7: Total	K10: Adults	K7: Adults	K10: Older adults	K7: Older adults
ΔPsychological distress score	Comparison group	Δmeans (95% CI)**	Δmeans (95% CI)**	Δmeans (95% CI)**	Δmeans (95% CI)**	Δmeans (95% CI)**	Δmeans (95% CI)**
Older adults	Adults	1.53* (0.92, 2.15)	1.06* (0.57, 1.56)				
Female	Male	0.11 (-0.78, 1.01)	0.004 (-0.72, 0.72)	1.09 (-0.42, 2.6)	0.33 (-0.87, 1.53)	0.52 (-0.66, 1.71)	-0.38 (-1.34, 0.57)
No education	Degree or equivalent	2.04* (0.66, 3.41)	1.58* (0.48, 2.69)	1.63* (0.09, 3.17)	1.11 (-0.13, 2.32)	2.54* (0.08, 4.99)	2.12* (0.15, 4.09)
	SSC or HSC pass	1.88* (1.01, 2.75)	1.16* (0.46, 1.85)	1.42* (0.42, 2.41)	0.76 (-0.03, 1.55)	3.34* (1.71, 4.96)	2.33* (1.03, 3.63)
	Secondary (6–10)	1.80* (0.96, 2.65)	1.35* (0.68, 2.03)	1.43* (0.45, 2.42)	1.30* (0.25, 1.81)	1.64* (0.09, 3.18)	1.18 (-0.06, 2.42)
	Primary (1–5)	.82* (0.19, 1.44)	0.24 (-0.26, 0.74)	0.62 (-0.23, 1.47)	0.05 (-0.63, 0.73)	0.76 (-0.13, 1.66)	0.22 (-0.52, 0.92)
Widowed	Married	1.95* (1.2, 2.71)	1.73* (1.12, 2.34)	0.91 (-0.67, 2.49)	0.87 (-0.39, 2.13)	2.48* (1.52, 3.43)	2.10* (1.33, 2.87)
	Unmarried/ never married	2.82* (0.98, 4.66)	2.21* (0.74, 3.69)	1.40 (-0.81, 3.6)	1.27 (-0.49, 3.02)	4.25 (-1.41, 9.92)	2.52 (-2.03, 7.06)
Student	House duties	1.37 (-0.76, 3.5)	0.93 (-0.78, 2.64)	-0.49 (-2.03, 1.06)	0.04 (-1.62, 1.69)	-1.78 (-7.43, 3.87)	2.19 (-2.35, 6.74)
	Farmer	0.54 (-1.69, 2.77)	0.34 (-1.45, 2.13)	-1.4 (-3.6, 0.81)	-0.79 (-2.58, 1.00)	-4.25 (-9.92, 1.41)	1.49 (-3.13, 6.1)
	Labour	1.58 (-0.61, 3.77)	1.26 (-0.5, 3.02)	0.28 (-1.8, 2.36)	0 (-1.75, 1.75)	3.18 (-2.48, 8.84)	2.77 (-1.96, 7.51)
	Business, Govt/ non-govt job	1.92 (-0.22, 4.07)	1.44 (-0.29, 3.16)	-1.35 (-3.6, 0.91)	0.64 (-1.07, 2.35)	2.3 (-3.45, 8.05)	2.02 (-2.56, 6.6)
	unable to work or retired	0.3 (-1.88, 2.47)	0.21 (-1.55, 1.94)	-0.43 (-2.62, 1.77)	-1.94 (-4.74, 0.86)	3.73 (-2.17, 9.63)	1.96 (-2.55, 6.48)
Insufficient funds some of the time	Insufficient fund all the time	-0.98* (-1.7, -0.23)	-0.71* (-1.31, -0.12)	-0.29 (-1.19, 0.61)	-0.20 (-0.92, 0.51)	-1.36* (-2.51, -0.2)	-0.99* (-1.91, -0.06)
	Medium (not good or bad)	-1.54* (-2.1, -0.97)	-1.31* (-2.77, -0.86)	-1.05*(-1.70, -0.39)	-0.93* (-1.45, -0.4)	-1.82*(-2.76, -0.89)	-1.49* (-2.24, -0.74)
	Sufficient funds all the time	-2.15 (-3.04, -1.25)	-2.20 (-2.92, -1.48)	-0.95 (-2.02, 0.11)	-1.29* (-2.14, -0.44)	-3.12 (-4.55, -1.7)	-2.96* (-4.1, -1.82)
Any health problem	No	1.18* (0.49, 1.87)	.97* (0.42, 1.53)	0.95 (-0.11, 2.02)	-0.05 (-0.72, 0.62)	3.12* (1.7, 4.55)	2.04* (1.15, 2.93)
On medication	No	0.21 (-0.45, 0.86)	0.42 (-0.11, 0.95)	-0.09 (-1.07, 0.88)	1.30* (0.56, 2.04)	1.30 (-0.05, 2.66)	-0.12 (-0.86, 0.61)
Current or past smoker	Never smoker	1.02* (0.39, 1.66)	.87* (0.36, 1.38)	-0.1 (-0.95, 0.74)	0.72* (0.11, 1.33)	2.44* (1.33, 3.54)	1.38* (0.56, 2.2)
	Smokeless tobacco	0.70 (-0.02, 1.42)	.68* (0.11, 1.26)	1.41* (0.48, 2.34)	0.97* (0.14, 1.81)	-0.51 (-1.42, 0.4)	.80* (0, 1.6)
Pouroshoba	Bashgram	1.36* (0.7, 2.03)	1.44* (0.9, 1.97)	2.18* (1.45, 2.92)	2.04* (1.45, 2.63)	0.4 (-0.74, 1.53)	0.72 (-0.19, 1.63)
	Tularampur	4.31* (3.61, 5.02)	3.73* (3.16, 4.29)	7.95* (7.09, 8.8)	6.81* (6.12, 7.49)	1.01 (-0.1, 2.11)	0.95* (0.07, 1.84)
	Vodrobila	3.26* (2.58, 3.95)	2.88* (2.32, 3.42)	3.90* (3.12, 4.68)	3.25* (2.63, 3.86)	2.55* (1.41, 3.69)	2.43* (1.51, 3.34)

*Indicates significant changes compared to the reference groups

**Difference in means (95% CI) adjusted for age, gender, level of education, marital status, socio-economic condition, occupation, smoking status and medical condition and geographic location.

## Discussion

Our study reported the prevalence of psychological distress, its severity and its associations with socio-demographic, lifestyle and medical conditions in a typical rural district in Bangladesh. Using the internationally validated K10 psychological distress measuring tool[27], we report that overall 52.5% of adults had any psychological distress—44% in people who were younger than 60 years of age and 62% in people who were older or equal to 60 years of age. Severe psychological distress was nine times more likely in older adults compared to younger adults. The study adds further evidence of high prevalence of psychological distress to a literature that was lacking current data for more than a decade.

The prevalence of psychological distress is higher in our study but the risk factors are found to be similar to previous studies. Results from a systematic review reported a wide variation of depressive symptoms in Bangladesh between 6.5 to 31.0% [20]. Choudhury et al. [31] reported the highest prevalence of 65% in a disaster affected area, with a higher percentage in females in Bangladesh. The prevalence of 6.5% was self-reported psychological distress for which data were collected from people of all ages from a single rural village in 1981. Prevalence of 31% was also self-reported psychological distress for which data were collected from people of age 13 years or older from a tertiary care hospital in 1975. The studies reported lower prevalence of psychological distress in rural areas, such as 6.5% to 16.5%, compared to 28% to 31.5% in urban areas [20]. Although our study suggested higher prevalence of psychological distress, the wide variation and lower prevalence in previous studies can be attributed to the use of different protocols, age ranges, assessment tools and different definitions of disease outcomes. The higher prevalence of psychological distress in urban areas is consistent with the previous studies in Bangladesh, and contradict those reported in Australian and US studies, which found higher prevalence of psychological distress in rural areas [11, 32].

Although there was no significant difference in overall prevalence of any psychological distress or severity of psychological distress between males and females, severity of psychological distress was found to be significantly higher in females after the age of 60 years, and these results are consistent with previous findings [33–37]. Higher prevalence of psychological distress multi-factorial in cause, and include lack of access to, and utilisation of, mental health services, stigma about mental health treatment, poor physical health, other medical conditions, disability and isolation [11, 38], and low SES [38, 39]. In our study, factors such as no education, being widowed, unable to work or being retired, and suffering from any medical condition were associated with higher prevalence of severe psychological distress, especially in older women. These findings are also consistent with the previous findings [34, 38]. This higher prevalence in females can also be explained by the fact that females are more susceptible to the established risk factors of psychological distress such as lower level of education and less engagement in any income generation activities in Bangladesh [40]. For example, our data showed that among people with no education, 57% were females and 43% were males, among widowed adults, 82% were females and 18% were males, among people having government or non-government jobs, 10% were females vs. 90% were males. Poor financial situation, especially in developing countries is also more frequent among females, which is reported to be associated with higher prevalence of psychological distress [41–43]. A study by the World Health Organisation reports that women who had experienced severe abuse, either physical, mental or sexual abuse, independently of the time period, also experienced more psychological distress in their older age [44]. Globally, sexual violence is experienced more by girls and women, and there is a strong association between being sexually abused in childhood and the presence of multiple mental health problems later in life [33, 44], although these findings can not be verified from the current study as these data were not collected. This indicates that being of female gender is not inherently associated with higher prevalence of psychological distress but the established risk factors are higher in females, which can be the main reason for higher prevalence of psychological distress, especially in older age women [40].

Insufficient funds most of the time (i.e. very poor) was associated with higher prevalence of psychological distress, both in males and females. Although Bangladesh has shown significant economic growth in recent years [45], 24.3% people still live under the poverty line and 12.9% live under an extreme poverty line, which is higher in rural areas than those in urban areas [46]. Gross family income not only impacts daily life, it is also associated with a low level of education, higher rate of unemployment, and poor or no medical facilities, and this leads to a severe psychological distress and poor quality of life. Interestingly, our data show a parabolic relationship between socio-economic status and psychological distress. That is, sufficient funds most of the time was also associated with higher prevalence of psychological distress compared to those with insufficient funds some of the time (poor to middle class). The causes of such an association in Bangladesh is difficult to explain. However, it is comparable to the global fact that the prevalence of depression and anxiety in some high income countries such as in France and United States is higher than that in lower-middle income countries such as in Brazil, India and China [47]. Cruza-Guet[48] reported a similar parabolic relationship between psychological distress and social support from a study of psychological distress among Hispanic elderly in USA, indicating both lower and higher level of facilities may cause more psychological distress than a medium level of facilities. Moreover, we have found that the prevalence of psychological distress was the lowest in people who are daily labourers and have claimed to have insufficient funds some of the time (poor), which indicates people with a reasonable income can lead a less distressful life than their relatively rich and very poor counterparts.

Although literature does exist on psychological distress and socio-economic condition in Bangladesh [20], the literature on smoking and psychological distress is very limited. The current study reports associations of smoking with psychological distress in older adults, both in males and females. From a cross-sectional study, Islam and Hossain [49] reported a similar association of smoking with higher prevalence of psychological distress, among the healthy adults university students Literature suggests that people with mental health problems have a higher prevalence of smoking than the general population [50]. At the same time, smoking has been reported to be linked to mental health conditions, either as a consequence or as a cause [43, 51]. Therefore, whether smoking causes psychological distress or vice versa is unknown and it cannot be explained from this cross-sectional study. Although data were collected from a rural upazila, some data were collected from the typical rural unions where no urban facilities exist and some data were collected from upazila headquarters which is an urban area. The current study shows that the prevalence of psychological distress was higher in urban pourashava compared to that in rural unions which is consistent with the previous studies [20].

The advantage of the present study is that it included a large sample with a wide age distribution and which collected data directly through a face-to-face interview from adults and older adults, with almost 50% female participants and 1147 people were 60 years of age or older. The study has reported the prevalence of and risk factors for psychological distress, both for adults and older adults separately with sufficient statistical power. The data was derived from a comprehensive questionnaire that included socio-demographic and medical factors along with smoking status reported either through smoking tobacco or use of SLT. Psychological distress was measured using the K10 questionnaire, which has been widely used. The psychometric properties were tested for the K10 questionnaire and a modified 7-item questionnaire was proposed. To check the robustness, both K10 and validated K7 were used to report the factors associated with the psychological distress score. Both K10 and K7 showed robust findings. It is also possible to report the associations of not only any psychological distress but also the severity of psychological distress with the risk factors due to the large sample size.

The study has a number of limitations. Firstly, data were collected from one district and thus the results may not be generalised at the national level. Whilst the sample was representative of the situation in Narail district, and the rural population is very homogenous in Bangladesh, the study’s results need to be extrapolated with caution to other rural parts of Bangladesh. Secondly, data on smoking and SLT use, and medical condition were based on self-report response. Reporting bias, reporting error and different perceptions about health conditions are very likely dependent on disease severity and the level of knowledge of the participants. Thirdly, the subjectivity in reporting availability of funds is not the most appropriate measure of SES. Therefore, the association of medical condition, SES and smoking with psychological distress need to be evaluated with caution. Fourthly, some key predictors such as history of mental health problems, exposure to violence, and other traumatic experiences were not included in the questionnaire.

## Conclusions

The prevalence of psychological distress in a rural area in Bangladesh was considerably high. Generally, the prevalence of mild psychological distress was similar in males and females but the prevalence of moderate and severe psychological distress in females showed a sharp increase after the age of 60 years. The risk factors associated with psychological distress in this sample included older age, low SES, being widowed, being unable to work and having a medical condition. The study suggests that hypertension, diabetes and other chronic diseases are closely linked and suggest a need for management through a common strategy.

## Supporting information

S1 Dataset Psychological distress.sav. (SAV)

S1 File Table 1 for Fig 1. (XLSX)

S2 File Table 2 for Fig 2. (XLSX)
